# Movi: a fast and cache-efficient full-text pangenome index

**DOI:** 10.1101/2023.11.04.565615

**Published:** 2023-11-05

**Authors:** Mohsen Zakeri, Nathaniel K. Brown, Omar Y. Ahmed, Travis Gagie, Ben Langmead

**Affiliations:** 1 Department of Computer Science, Johns Hopkins University; 2 Department of Computer Science, Dalhousie University

## Abstract

Efficient pangenome indexes are promising tools for many applications, including rapid classification of nanopore sequencing reads. Recently, a compressed-index data structure called the “move structure” was proposed as an alternative to other BWT-based indexes like the FM index and r-index. The move structure uniquely achieves both O(r) space and O(1)-time queries, where r is the number of runs in the pangenome BWT. We implemented Movi, an efficient tool for building and querying move-structure pangenome indexes. While the size of the Movi’s index is larger than the r-index, it scales at a smaller rate for pangenome references, as its size is exactly proportional to r, the number of runs in the BWT of the reference. Movi can compute sophisticated matching queries needed for classification – such as pseudo-matching lengths – at least ten times faster than the fastest available methods. Movi achieves this speed by leveraging the move structure’s strong locality of reference, incurring close to the minimum possible number of cache misses for queries against large pangenomes. Movi’s fast constant-time query loop makes it well suited to real-time applications like adaptive sampling for nanopore sequencing, where decisions must be made in a small and predictable time interval.

## 1 Introduction

Pangenome indexes are promising tools for aligning and classifying sequencing reads with respect to large sets of similar reference sequences. While many existing tools are k-mer based [1, 2], others use flexible full-text indexes enabling arbitrary-length pattern matching queries, like the FM-index [3, 4] and r-index [5, 6]. The FM-index and r-index are full-text indexes that facilitate matching via “backward search.” The r-index can also find maximal exact matches (MEMs) and matching statistics using the MONI algorithm [7]. Unlike the FM-index, the r-index is run-length compressed, allowing the index grow proportionally to the amount of *distinct* sequence in the reference (pangenome) rather than the total sequence.

In practice, the r-index comprises a collection of data structures such as bitvectors and wavelet tries. A single query – such as a single backward-search step – usually requires memory accesses to many disparate places on these structures. The number and unpredictability of these accesses leads to cache misses, i.e. pauses wherein the processor must wait for portions of data structures be moved from main memory into more proximate cache memories. Even when the overall time required by an index query is theoretically constant, the latency incurred by cache misses can be large, making queries slow in practice. Also, variability in the number of cache misses incurred per query leads to fluctuating latency across queries. Overall, the effect is to make queries slow with high variability.

The Move structure was introduced by Nishimoto and Tabei in 2021 [8]. Like the FM-index and r-index, it is a full-text index based on the Burrows Wheeler Transform (BWT). It achieves both O(r) space usage and O(1) (constant) time for LF mapping queries. This combination has not achieved by other indexes; e.g. the r-index can achieve one or the other but not both. Another key advantage of the move structure is that it consists entirely of a single table. Move structure queries need only perform a limited only of accesses to this table, incurring few – usually just one or two – cache misses per query. That is, move structure queries have excellent locality of reference. This should lead to queries that are faster and with more predictable latency compared to alternatives like the r-index. While past studies have shown some of the move structure’s computational trade-offs relative to r-index [9], no studies have investigated these advantages related to speed and locality of reference.

Here we introduce Movi, a pangenome full-text index based on the move structure. Movi is much faster than alternative pangenome indexes like the r-index. We also measure Movi’s cache characteristics and show that, as hypothesized, queries achieve a small (nearly minimal) number of cache misses. We demonstrate that Movi can implement the same algorithms as alternative pangenome tools like [5]. Finally, we show that despite having a larger size compared to other pangenome indexes, Movi grows more slowly than other pangenome indexes as genomes are added.

In short, Movi is the fastest available tool for full-text pangenome indexing and querying, and our open source implementation enables its application in various classification and alignment scenarios, including in speed-critical scenarios like adaptive sampling for nanopore sequencing.

## 2 Methods

### 2.1 Burrows Wheeler Transform, FM-index and r-index

**Figure 1:**
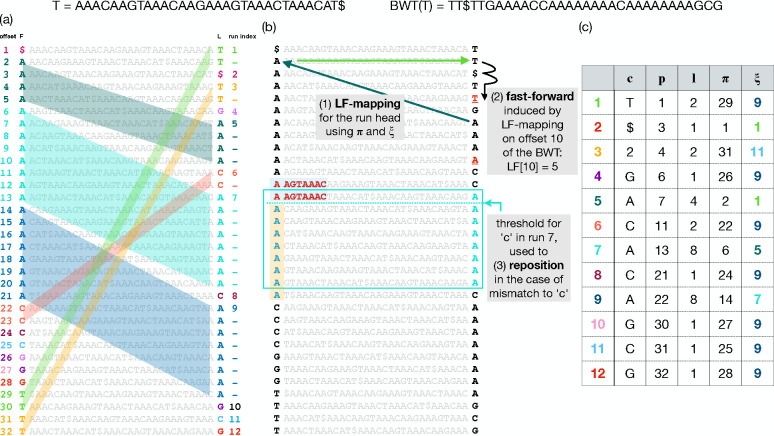
T and BWT(T) are shown at the top. (a) BWM(T), consisting of T’s distinct rotations sorted. The leftmost column is called F and the rightmost column is BWT(T), also called L. BWT runs in L are given distinct colors. The LF-mapping maps these runs to same-letter stretches in L. This is illustrated using matching colors and, in the case of multi-character runs, by parallelograms connecting BWT characters to their LF counterparts. (b) Arrows at the top illustrate how a move-structure query for LF[10] results in one LF step (green arrows) followed by two fast forward steps (black arrow). Below, the light blue arrows and boxes illustrate how a threshold facilitates the “repositioning” process, where a mismatch between the BWT character (“A”) and a “C” in the query causes a repositioning to the nearest offset above ending in “C,” chosen because it has a longer longest common prefix with the initial row than the candidate below. The threshold, depicted with a light blue horizontal dotted line denotes the point above which rows have a longer LCP with the next C-terminated row above, but rows below have a longer LCP with the next C-terminated row below. (c) Each BWTrun is represented as a row in the move structure table; c is the run character, ℓ is the run length, p is the offset of the run with respect to the beginning of the BWT, π is LF[p], and ξ is the index of the run containing offset π.

The Burrows Wheeler Transform (BWT) is a reversible permutation that reorders the characters of a string T according to the lexicographical order of their right contexts in T. Beginning with T of length n, we append a terminal symbol $ that does not appear elsewhere in T and is lexicographically smaller than T’s other characters. T[i] denotes the character at 1-based offset i and T[i..n] denotes a suffix starting at i. BWT(T) permutes T’s characters so that T[i] comes before T[j] in BWT order if and only if T[i+1…n]<T[j+1..n]. The BWT tends to reveal repetitiveness in T. Repetitive portions of T yield long “runs” in BWT(T) where a run is a maximal-length substring consisting of a character repeated. This is illustrated in Figure 1a, where runs of lengths up to 8 are visible in the last column of the matrix.

Figure 1a and Figure 1b show two copies of a Burrows-Wheeler Matrix or BWM. Rows of the BWM consist of all distinct rotations of the string T, ordered lexicographically. BWT(T) is the last column of BWM(T). The first and last columns of the BWM are related by the Last-to-First mapping (“LF-mapping”) [10]. The LF-mapping says that the i^th^ occurrence of a character c in the last column of the BWM corresponds to the same text occurrence as the i^th^ occurrence of c in the first column. Some of these relationships are illustrated using parallelograms in Figure 1a. The LF-mapping also gives a way to navigate through the text T. Note that if BWT permutation maps T[j] to BWT[i], then LF[i] gives the BWT index of T[j–1] (or T[n] if j=1). So the LF-mapping allows for right-to-left movements with respect to T, a fact used in pattern-matching queries.

The FM-index is a data structure based on BWT(T) enabling fast and efficient computation of the LF-mapping and related queries. It consists of BWT(T) as well as succinct data structures for storing and querying character ranks within BWT(T). In typical implementations, it grows linearly with the text: O(n).

When T is repetitive, the number of BWT runs (r) is much smaller than the text length (n). The r-index [11] exploits this by representing the BWT in a run-length-compressed fashion. This version is called the RLBWT. The i^th^ run, denoted RLBWT [i], is represented by RLBWT [i].c, the character repeated in the run, and RLBWT [i].n, its length. Additional data structures enable efficient computation of the LF-mapping without having to decompress the RLBWT. The data structures making up r-index fit in O(r) space total.

### 2.2 The Move Data Structure

#### LF Mapping

When T is a repetitive pangenome, the LF-mapping tends to map consecutive stretches of BWT characters to other consecutive stretches (Figure 1a). The move structure exploits this to compute LF-mapping with a simple procedure. The move structure consists of a table (M) with rows corresponding to BWT runs (Figure 1c). To aid LF-mapping, column M.π stores the LF-mapping of the run head, i.e. M.π=LF[M.p]. To compute the LF-mapping at any offset in run index i, we can begin by following M[i].π. This will either jump to the correct run, or to a run preceding the correct one.

Given M, a BWToffset j, and a run index i, we compute LF[j] by adding j’s offset into the current run (j−M[i].p) to the run head’s LF-mapping:

LF[j]=M[i]⋅π+(j−M[i]⋅p)

This involves simple arithmetic on i,j,M[i].π and M[i].p. It does not involve bitvectors or wavelet-tree queries. Only the accesses to M[i] might require accessing main memory. An illustration of how memory accesses induced by move structure queries differ than those by r-index is shown in Figure S2

Note that an input to this computation is i, the current run index. To chain multiple LF-mapping queries together, as is needed for matching queries, we must update not only the BWT offset but also the BWT run index. As a step toward this goal, $M\left[i\right].\xi$ stores the index of the run containing LF[M[i].π]. However, the run containing LF[M[i].π] may not also contain LF[j]. I.e. it is possible that LF[j]−M[M[i].ξ]⋅p>M[M[i].ξ].ℓ. After jumping to M[M[i].ξ], we may additionally need to advance through the runs until finding the smallest run index $i^{\prime }>i$ such that $M\left[i^{\prime }\right]\cdot p\leq LF[j]<M\left[i^{\prime }+1\right].p$. We call this the “fast-forward” or “ff” procedure, illustrated in Figure 1b (top) and detailed by Algorithm S1 in supplementary materials. Using that algorithm, we update both i and j in each LF-mapping step:

$$i^{\prime }\leftarrow ff(M,i,j)\;j^{\prime }\leftarrow M[i]\cdot \pi +(j-M[i]\cdot p)$$

#### Constant-time LF

Nishimoto and Tabei gave a procedure for splitting some BWT runs into shorter subruns to achieve a constant upper bound on the number of fast-forwards required for any LF-mapping [8]. The procedure works with a parameter d such that, after splitting runs, the number of fast-forwards per LF-mapping query is less than 2d while adding at most $\frac{r}{d-1}$ additional runs to the table. The overall number of runs is still O(r) after splitting. In practice, the procedure splits only a fraction of the original runs.[12]

With the exception of the jump induced by following LF[M[i].π], all the memory accesses described here are sequential. The LF[M[i].π] step is unpredictable, possibly needing to access a not-recently-accessed location in memory, likely incurring a cache miss. That said, for a chain of several LF-mapping queries, only one expensive memory access is needed per query.

Since the information about the exact BWT offset of the exact matches is not required for computing pseudo mapping lengths, Movi avoids storing both p and π in the table. Instead, Movi collapses those fields into a single relative offset, as previously implemented by Brown et al [9].

### 2.3 Computing pseudo matching lengths (PMLs) with move structure

#### Matching statistics and pseudo matching lengths

Matching statistics (MS) are a useful summary of sequence similarity, used in sequence classification tasks and for computing other similarity features like Maximal Exact Matches (MEMs). Given a text T[1..n] of length n and a pattern P[1..m] of length m, the matching statistics of P against T are defined as an array MS[1..m] of length m, where each MS[i] stores the length of the longest prefix of P[i..m] that occurs in T.

Bannai et al. [13] described a 2-pass procedure for computing matching statistics using the r-index and an auxiliary “thresholds” structure. Later, Rossi et al. [7] gave an efficient procedure for computing the thresholds. Later, Ahmed et al. [5] introduced a modified 1-pass version of the procedure that computes a vector of Pseudo Matching Lengths (PMLs), which roughly approximate the lengths in MS. While PMLs contain somewhat less information than MSs – e.g. they cannot be used to exactly compute MEMs – finding PMLs is faster, can be performed in single pass over the query, and requires neither a suffix-array sample nor a random-access structure over T. In practice, PMLs are similar to MSs in their ability to classify sequences.

The MONI algorithm starts at an arbitrary offset in the BWT, then considers each character of the query sequence in right-to-left order. Say we are currently at offset j in BWT and are examining character P[i]. The algorithm first tests if P[i]=BWT[j]. If they are equal, we call this “case 1.” For case 1, the algorithm performs an LF-mapping step and moves on to the next character:

$$i^{\prime }\leftarrow i-1\;j^{\prime }\leftarrow LF[j]$$

The LF-mapping uses the strategy we discussed above, which includes the fast-forward procedure. If P[i]≠BWT[j], we call this “case 2.” For case 2, we cannot simply use LF[j] as our next offset; rather we must “reposition” to a nearby offset $j^{r}$ such that $P[i]=BWT\left[j^{r}\right]$. We let $j^{r}$ equal one of two choices: the greatest $j^{\text{up}}$ such that $j^{\text{up}}<j$ and $BWT\left[j^{\text{up}}\right]=P[i]$, or the smallest $j^{\text{dn}}$ such that $j^{\text{dn}}>j$ and $BWT\left[j^{\text{dn}}\right]=P[i]$. Whether we choose $j^{up}$ or $j^{dn}$ is determined by first consulting the thresholds structure of Bannai et al. [13]. Once we have repositioned, we proceed using the same update rule as above, substituting $j^{r}$ for j.

Instances where we can apply the simpler case 1 update rule correspond to instances where an existing match is being extended by 1 character, causing the matching statistic to increase by one. Instances where we apply case 2 might correspond to an extension or might or might not correspond to an extension. The MS algorithm from MONI is capable of distinguishing these two subcases of case 2. The PML algorithm of SPUMONI is not capable of this, instead resetting the match length to 0 when it reaches an instance of case 2. Details about the PML computation procedure is shown by Algorithm S2 in supplementary materials.

#### movi’s repositioning

Movi uses two distinct strategies for finding and moving to the run containing $j^{r}$. By default Movi, scans from run to run (either upward or downward, depending on the threshold) until a run with a matching character is detected. This leads to an unbounded number of memory accesses, though these accesses are sequential. In its Movi-constant mode, Movi instead stores explicit pointers to the $j^{r}$-containing runs for each characters of the DNA alphabet. It stores two such sets of pointers, one for when the threshold points upward and one for when it points downward, leading to a total of six additional pointers being stored in each move structure run.

**Figure 2:**
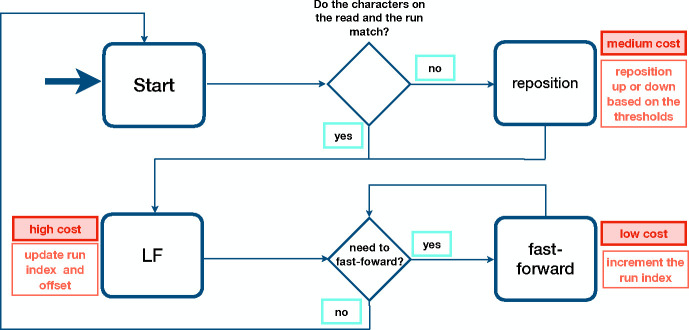
Pseudo length computation with Movi. The typical cost associated with each type of memory access is described. Higher cost accesses are those that tend to move long distances to memory addresses that have not been used recently.

In short, there are three types of operations performed by Movi during the PML computation; (1) jump to the run potentially containing the LF-mapping destination, (2) fast-forward to the run that contains the LF-mapping destination, and (3) reposition to the run containing a matching character in the case of a mismatch. These three operations are illustrated in a state diagram Figure 2 (and with more details in supplementary materials Figure S1). The first type (1) is inevitable for every new LF-mapping and occurs exactly once per base, it also has the highest cost in terms of latency and cache-miss. Type (2) and (3) are cheaper operations with fast-forwards being the cheapest. For Movi-default, the number of iterations for each per base is not guaranteed to be constant theoretically and we evaluate an extensive explorations of their behaviour in Section 3.2. These operations are guaranteed to be bounded in Movi-constant by employing the splitting algorithm and including the extra pointers for the destination of repositioning jumps in the case of mismatches.

### 2.4 The Movi software

Movi supports two modes of operation. The first mode, called Movi-default, is fast and simple but lacks the constant-time LF-mapping query guarantee. The second mode, called Movi-constant, uses the splitting to create a move structure that has a constant-time LF-mapping guarantee. Further, Movi-constant uses a constant-time version of the repositioning step, allowing its inner loop to be fully constant-time, regardless of whether it involves LF-mapping steps and/or repositioning. This comes at the cost of additional space, since (a) the move structure that results from the splitting procedure has more runs and is therefore somewhat larger than the unsplit move structure, and (b) the constant-time repositioning step requires that we pre-compute upward and downward jump distances and store them in the move structure table.

To build the Burrows Wheeler Transform, Movi uses the prefix-free parsing (PFP) algorithm of Boucher et al [14], which is particularly efficient for building the BWT of a highly repetitive text such as a pangenome. The algorithm also integrates Rossi et al’s [7] approach for computing thresholds for repositioning.

Movi is implemented in C++. It is GPL3-licensed open-source software available from https://github.com/mohsenzakeri/movi. It depends on both the prefix-free parsing implementation from the pfp_thresholds repository ^1^ and the run splitting implementation from the r-permute library ^2^.

## 3 Results

We measured Movi’s speed and cache characteristics relative to the related SPUMONI approach as well as to other approaches that use the FM Index (Bowtie 2), a pangenome k-mer index (Fulgor) or other approaches that achieve compression (minimap2). We measure the predictability of Movi’s innermost loop, to assess its utility for real-time data processing applications. Finally, we explores how Movi’s index scales when applied to pangenomes using the data available from the Human Pangenome Reference Consortium (HPRC)[15]. All experiments were run on 3 GHz Intel Xeon Gold Cascade Lake 6248R CPU with 1.5TB DDR4 2933MHz memory.

### 3.1 Computing pseudo matching lengths for a mock community

We first measured the move structure’s efficiency for computing pseudo matching lengths (PMLs), an approximation of matching statistics previously shown to be useful for classification tasks, including adaptive sampling [5, 6]. We compared Movi’s default and constant modes to SPUMONI in terms of index size and query time. We ran the tools on the Zymo High Molecular Weight Mock Microbial Community (SRR11071395) previously used to evaluate Uncalled [16].

For further context, we also evaluated the FM-index based tool Bowtie2, the minimizer and hashtable-based tool minimap2, and the colored compacted de-bruijn graph-based tool Fulgor. Note that these tools differ in what they actually compute, with Bowtie2 and minimap2 generating full read alignments, and Fulgor producing pseudo-alignments. The sample consists of about 800K long reads sequenced by Oxford Nanopore Techonologies (ONT) with the average length of 15K bases.

For all tools, the index consisted of all the complete reference genomes of 7 bacteria species (Bacillus subtilis, Enterococcus faecalis, Escherichia coli, Listeria monocytogenes, Pseudomonas aeruginosa, Salmonella enterica, and Staphylococcus aureus). These were all obtained from RefSeq database [17].

Table 1 shows the size of the indexes built by all the tools as well as the time required for querying all the reads. We first compared the computational requirements of Movi-default to SPUMONI. We observed that Movi-default was 12 times faster than SPUMONI , but its index was 4.7 times larger than SPUMONI’s. Movi-constant was both slower and had a larger index compared to Movi-default; as we show later, however, the Movi-constant mode benefits from more predictable performance across inner-loop iterations.

Fulgor had both a smaller index and a relatively fast query time compared even to Movi, taking only about 1.6 times the amount of time as Movi-default. Fulgor’s full index takes about 3 GB, about one third the size of Movi-default’s 8.5 GB index. On the other hand, the two tools output different results, with Movi outputting pseudo-matching lengths and Fulgor outputting pseudo-alignment information. Further, Fulgor is k-mer based and requires pre-selection of a set k-mer length, whereas Movi is a full-text index. Movi-default is the fastest overall and provides an advantageous trade for applications that benefit from the flexibility of a full-text index, e.g. adaptive sampling.

Bowtie2 and minimap2 are not perfectly comparable to Movi since they produce full read alignments. Further Bowtie2 is designed for use with short reads, not the long nanopore reads assessed here. For that reason, we omitted Bowtie2 from the speed comparison. Minimap2 took about 33 times longer to align the reads, while also using 16 threads (compared to 1 thread for the other tools). Its index was also 8 times larger than Movi-default’s. So although minimap2 is able to produce full and accurate alignments for the nanopore reads, Movi provides a useful combination of speed and memory efficiency for applications, such as classification, where pseudo matching lengths provide sufficient power.

Finally, we compared the PMLs generated by Movi (both modes) against those computed by SPUMONI. Using the diff tool, we found that Movi and SPUMONI generated identical PMLs, as expected.

### 3.2 Predictability of queries with Movi

Because of its simple tabular form, we hypothesized the move structure would exhibit superior cache characteristics compared to SPUMONI.

We used the “Cachegrind^3^” profiler to measure the cache misses incurred by Movi and SPUMONI when computing PMLs for the same Zymo sample used in the previous section. Specifically, we measured misses in the “last-level” cache, i.e. the final level of cache before main memory. These are the cache misses that require the longest pauses.

**Figure 3:**
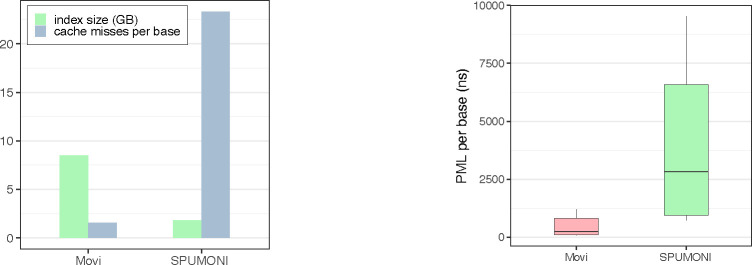
Comparisons of Movi-default and SPUMONI in terms of query speed and predictability.

Figure 3a shows the number of cache misses per base. We observed that SPUMONI incurred more than 14 times as many cache misses per base compared to Movi. The reduced cache miss rate of Movi came at the cost of a larger index. We also observed that the time required for each iteration of the inner loop was both smaller and less variable for Movi compared to SPUMONI .

To assess the latencies of LF-mapping executed by SPUMONI and Movi more precisely, we employed the chrono high-resolution clock in C++ to make nanosecond-level latency measurements for their inner loops. The distribution of these latencies is visualized as boxplots in Figure 3b. We observed that iterations of the Movi inner loop were about 11.5 times faster than those of SPUMONI (comparing means). The 99th percentile of the latencies observed for Movi’s inner loop was smaller than the 1st percentile latency observed for SPUMONI’s inner loop.

**Figure 4:**
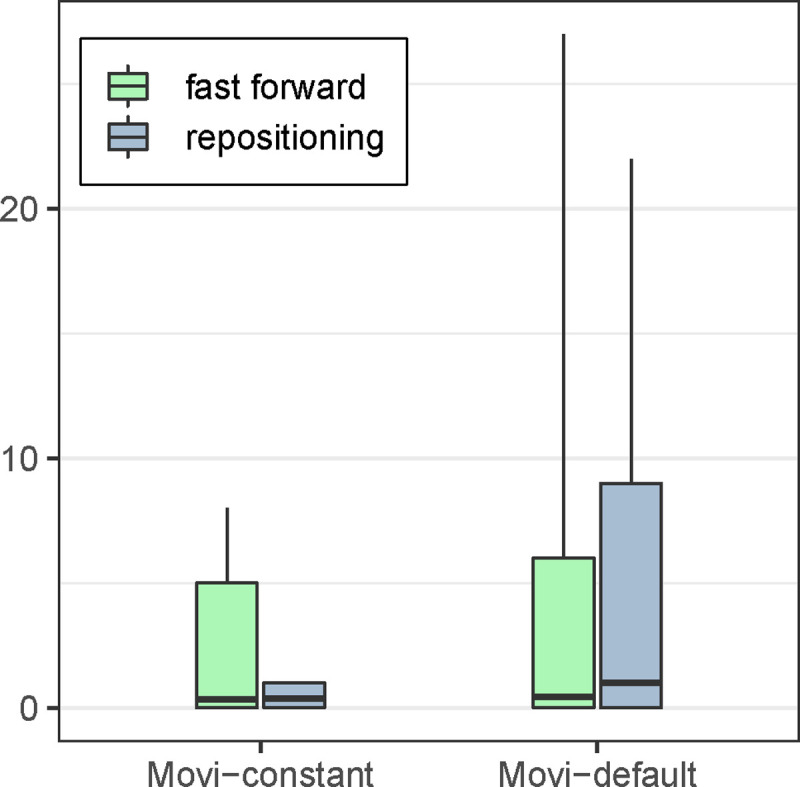
The number of fast-forwards and repositioning scans in each mode of Movi. The Movi constant is guaranteed to have always constant number of memory access per LF-mapping. Boxes extend from 1st to 99th percentiles and whiskers extend from 0.1^th^ to 99.9^th^. Horizontal line denotes mean. The median for all is 0.

Besides variability in inner loop performance due to cache misses, we also measured the number of fast-forward iterations and repositioning scans in each of Movi’s modes. These were discussed in Section 2.3. As expected, the number of operations was bounded by a small constant for Movi-constant. For Movi-default, the number of operations varied much more, as seen in Figure 4. Detailed statistics are presented in supplementary materials Table S1. While we earlier observed that Movi-default was faster than Movi-constant on average, here we saw that Movi-constant’s inner loop performed a smaller and more predictable number of operations, which is advantageous in situations where the algorithm must keep up with the output of an instrument in real-time. However, the average number of fast-forwards performed in Movi-default’s loop compared to Movi-constant’s was only about about 1.2 times greater, and the average number of repositioning scans was only about 2.5 times greater. The fact that Movi-default is still faster than Movi-constant despite this difference is likely because of the fact that Movi-constant requires a larger index, which in turns incurs more cache misses overall.

### 3.3 Extrapolation to nanopore throughputs

Using per-base speeds measured for the Zymo input data (presented in Table 1), we extrapolate to measure their ability to analyze nanopore sequencing data in a real-time adaptive sampling context. We assume that the sequences are base-called immediately. Considering that the sequencing speed of each nanopore of an Oxford Nanopore (ONT) instrument is 420 base pairs per second, SPUMONI ‘s speed is sufficient to simultaneosuly handle 904 channels (pores) at once. On the other hand, Movi can handle 11,071 simultaneous channels, surpassing the total number of channels in the largest flow cell available for the PromethION device: 2,675 channels^4^. Assuming perfect linear scaling, about 12 Movi threads would be sufficient to handle the output of 48 PromethION flowcells running simultaneously.

### 3.4 The Move structure scales well for the pangenome data

We next evaluated the scalability of Movi using human genome haplotype assemblies from the Human Pangenome Reference Consortium (HPRC)[15]. We selected various numbers of haplotypes, ranging from 1 to 94, which includes all available haplotypes. We measured the overall size and scalability of Movi’s indexes (based on the move structure) when compared to SPUMONI (based on r-index) and Fulgor (based on colored compacted de-bruijn graph). Note that Fulgor’s index also stores “color” information (associating k-mers with haplotypes), which is not a type of information stored in the Movi or SPUMONI indexes. We used k=31 and m=19 when building the Fulgor indexes.

**Figure 5:**
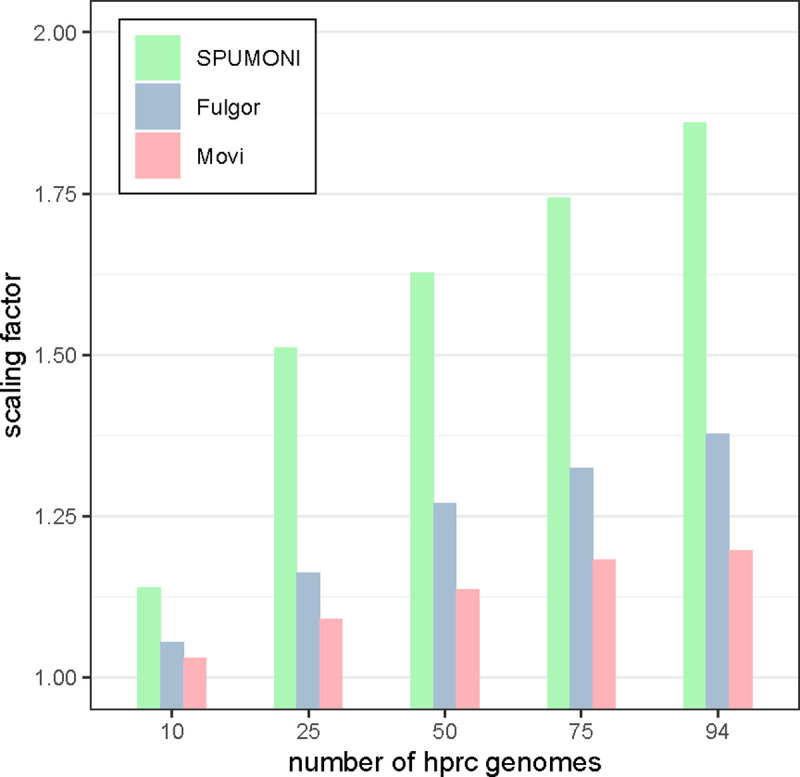
The scaling factor is computed by dividing the size of each tool’s index by the size of the index of that tool built over 5 hprc genomes. All tools have small scaling factors for pangenomes. While Movi’s index is the largest compared to the other two, it has the best scaling factor for any number of hrpc genomes.

We measured each tools’ ability to scale to larger pangenomes in Table 2. As a baseline for measuring scalability, we reported the number of distinct k-mers in the input according to Fulgor’s stats command (“kmer-count” column). As a second baseline, we also reported the number of runs in the BWT according to Movi (“r” column). As seen in Figure 5, the size of the 94-haplotype indexes were less than 2 times the size of the 5-haplotype indexes for all three tools. Movi exhibited the best scaling factor, with its 94-haplotype index using about 1.2 times the space as its 5-haplotype index. The 94-haplotype index for Fulgor and SPUMONI used 1.38 and 1.86 times the space as their 5-haplotype indexes respectively. This highlights the advantages of compressed indexes, including full-text indexes, when indexing large pangenomes.

Our results also demonstrated that the size of Fulgor’s index is considerably smaller than both SPUMONI and Movi’s. We note that Fulgor’s index includes both k-mer mapping and color class information, i.e. information about which k-mers occur in which haplotypes. In this HPRC experiment, the color class information accounts for a relatively small portion of the index, as the number of colors is limited. Running the ‘stats’ command in Fulgor for the indexes created in Table 2 demonstrates that only between 1% to 5% of the index size is attributed to storing the color information.

We also evaluated query speed for each tool using a simulated long read sample and a “combined” sample, consisting of both simulated reads and real reads from a human gut sample. This allows us to measure performance in a scenario where many input reads do not have a long match to the reference pangenome. The results are shown in Table S2 and are similar to those presented in Section 3.1, with Movi being fastest followed by Fulgor and SPUMONI .

## 4 Discussion and Future work

We introduced Movi, a cache-efficient, scalable tool for pangenomic indexing and read classification. Movi’s index is based on the move structure which is a full-text index with a scaling factor superior to competing approaches like SPUMONI and Fulgor. Movi is extremely fast, primarily due to its excellent locality of reference which in turn minimizes cache misses. Movi’s rapid and predictable query speed makes it well suited to applications like nanopore adaptive sampling. Movi can process the base-called output of a fully loaded PromethION using 12 threads.

The move structure’s simple tabular structure suggests simple ways to partition and distribute it across nodes of a computer cluster while minimizing inter-node communication. It can simply be divided into separate, contiguous chunks of rows, which can then be distributed. Execution of a pattern-matching query will require some jumps between nodes (i.e. a longer-distance LF query), but will frequently require only sequential or nearby jumps (fast-forwards and repositions) that do not require moving across nodes. This provides a much more favorable substrate for distributed computing compared to r-index, which is characterized by complex and unpredictable memory accesses.

Another key advantage of our full-text indexing approach is that it does not require the user to select any key parameters ahead of time. This is in contrast to k-mer based or minimizer-based approaches, for which the user must be aware of the potential pitfalls of choosing suboptimal parameters.

A key limitation of Movi is the fact that the table M itself is large compared to all the other tools assessed here (besides minimap2). In the future, it will be important to reduce the footprint of Movi’s index. This could be accomplished, for instance, by adopting the minimizer digestion strategy of SPUMONI 2 [6]. Another space-saving measure could be to losslessly compress the move structure using, e.g., the columnar compression strategies investigated by Brown et al. in 2022 [9].

It will also be important to expand Movi’s applicability to a broader range of query types. For instance, Movi could be adapted to handle multi-class classification by augmenting the index with suffix array or “document” information[18].

While Fulgor [19] optimizes space and time by capitalizing on long unitigs and explicitly storing the corresponding strings, we can adopt a similar strategy by leveraging substructures within the BWT. One such approach is to enhance query efficiency by reordering the BWT rows. This technique can be seamlessly integrated into Movi, enabling further cache efficiency and greater speed. By incorporating reordering, Movi has the potential to achieve even greater query performance.

## Supplementary Material

Supplement 1

## Figures and Tables

**Table 1:** Indexes are built over all available complete genomes of 7 bacteria from RefSeq database. The size of the fasta file including the recursive complement is 67 GB. The number of long reads in the sample is 800K.

Tool	index	full text	query type	document data (color)	size (GB)	query (hh:mm:ss)

Movi-default	move	yes	PML	no	8.5	00:45:22
Movi-constant	move	yes	PML	no	14	00:57:28

SPUMONI	r-index	yes	PML	no	1.8	09:20:55
Bowtie2	FM-index	yes	alignments	yes	12×2 + 40+	-
Minimap2	k-mer	no	alignments	yes	68	24:56:01*
Fulgor	k-mer	no	pseudo-alignments	yes	0.65 + 2.34^†^	01:11:51

* The minimap2 is run with 16 threads unlike other tools which are run with a single thread.

+ 12x2 shows the size of two FM-index in the Bowtie2’s index (the forward and reverse strand).

† The size of the Fulgor’s index is breakdown into two parts; the size of the k-mer set is 0.65 GB and the size of the index related to the document (color) information is 2.34 GB.

**Table 2:** Indexes are built over different number of hprc assemblies: 1, 5,10, 25, 50, 75, 94 (all)

reference	fasta (GB)	kmer-count (×10^9^)	Fulgor (GB)	r (×10^9^)	SPUMONI (GB)	Movi (GB)

hprc 1	2.9	2.50	3.1	3.33	6	62
hprc 5	15	2.70	3.7	3.53	8.6	66
hprc 10	29	2.79	3.9	3.65	9.8	68
hprc 25	74	2.94	4.3	3.84	13	72
hprc 50	174	3.06	4.7	4.02	14	75
hprc 75	214	3.13	4.9	4.14	15	78
hprc 94	268	3.19	5.1	4.24	16	79

## Footnotes

1
https://github.com/maxrossi91/pfp-thresholds

2
https://github.com/drnatebrown/r-permute

3
https://valgrind.org/docs/manual/cg-manual.html

4
https://nanoporetech.com/products/specifications

## References

[1] Wood D. E. & Salzberg S. L. Kraken: ultrafast metagenomic sequence classification using exact alignments. Genome Biology 15, 1–12 (2014). 10.1186/gb-2014-15-3-r46 PMC4053813 24580807

[2] Wood D. E., Lu J. & Langmead B. Improved metagenomic analysis with Kraken 2. Genome Biology 20, 257 (2019). 31779668 10.1186/s13059-019-1891-0 PMC6883579

[3] Kim D., Song L., Breitwieser F. P. & Salzberg S. L. Centrifuge: rapid and sensitive classification of metagenomic sequences. Genome Research 26, 1721–1729 (2016). 27852649 10.1101/gr.210641.116 PMC5131823

[4] Menzel P., Ng K. L. & Krogh A. Fast and sensitive taxonomic classification for metagenomics with Kaiju. Nature Communications 7, 11257 (2016). 10.1038/ncomms11257 PMC4833860 27071849

[5] Ahmed O. Pan-genomic matching statistics for targeted nanopore sequencing. iScience 24, 102696 (2021). 34195571 10.1016/j.isci.2021.102696 PMC8237286

[6] Ahmed O. Y., Rossi M., Gagie T., Boucher C. & Langmead B. Spumoni 2: improved classification using a pangenome index of minimizer digests. Genome Biology 24, 122 (2023). 37202771 10.1186/s13059-023-02958-1 PMC10197461

[7] Rossi M., Oliva M., Langmead B., Gagie T. & Boucher C. MONI: A Pangenomic Index for Finding Maximal Exact Matches. Journal of Computational Biology 29, 169–187 (2022). 35041495 10.1089/cmb.2021.0290 PMC8892979

[8] Nishimoto T. & Tabei Y. Optimal-time queries on bwt-runs compressed indexes. In 48th International Colloquium on Automata, Languages, and Programming (ICALP 2021), vol. 198, 101 (Schloss Dagstuhl–Leibniz-Zentrum fur Informatik, 2021).

[9] Brown N. K., Gagie T. & Rossi M. RLBWT Tricks. In Schulz C. & Uçar B. (eds.) 20th International Symposium on Experimental Algorithms (SEA 2022), vol. 233 of Leibniz International Proceedings in Informatics (LIPIcs), 16:1–16:16 (Schloss Dagstuhl – Leibniz-Zentrum für Informatik, Dagstuhl, Germany, 2022). URL https://drops.dagstuhl.de/opus/volltexte/2022/16550.

[10] Ferragina P. & Manzini G. Indexing compressed text. Journal of the ACM (JACM) 52, 552–581 (2005).

[11] Gagie T., Navarro G. & Prezza N. Optimal-time text indexing in bwt-runs bounded space. In Proceedings of the Twenty-Ninth Annual ACM-SIAM Symposium on Discrete Algorithms, 1459–1477 (SIAM, 2018).

[12] Brown N. Bwt-runs compressed data structures for pan-genomics text indexing (2023).

[13] Bannai H., Gagie T. & I, T. Refining the *r*-index. Theoretical Computer Science 812, 96–108 (2020).

[14] Boucher C. Prefix-free parsing for building big bwts. Algorithms for Molecular Biology 14, 1–15 (2019). 31149025 10.1186/s13015-019-0148-5 PMC6534911

[15] Liao W.-W. A draft human pangenome reference. Nature 617, 312–324 (2023). 37165242 10.1038/s41586-023-05896-x PMC10172123

[16] Kovaka S., Fan Y., Ni B., Timp W. & Schatz M. C. Targeted nanopore sequencing by real-time mapping of raw electrical signal with uncalled. Nature Biotechnology 39, 431–441 (2021). 10.1038/s41587-020-0731-9 PMC8567335 33257863

[17] O’Leary N. A. Reference sequence (refseq) database at ncbi: current status, taxonomic expansion, and functional annotation. Nucleic Acids Research 44, D733–D745 (2016). 26553804 10.1093/nar/gkv1189 PMC4702849

[18] Ahmed O., Rossi M., Boucher C. & Langmead B. Efficient taxa identification using a pangenome index. Genome Research gr–277642 (2023). 10.1101/gr.277642.123 PMC10538492 37258301

[19] Fan J., Singh N. P., Khan J., Pibiri G. E. & Patro R. Fulgor: A fast and compact k-mer index for large-scale matching and color queries. bioRxiv 2023–05 (2023). 10.1186/s13015-024-00251-9 PMC10810250 38254124

[20] Ono Y., Asai K. & Hamada M. Pbsim2: a simulator for long-read sequencers with a novel generative model of quality scores. Bioinformatics 37, 589–595 (2021). 32976553 10.1093/bioinformatics/btaa835 PMC8097687

