# Supplementary material for: Movi: a fast and cache-efficient full-text pangenome index
Source: bioRxiv. 2024 Feb 15:2023.11.04.565615. Originally published 2023 Nov 5. Preprint. [Version 2] doi: 10.1101/2023.11.04.565615 (PMC10635132; doi:10.1101/2023.11.04.565615)
Supplement: Supplement 1 [file NIHPP2023.11.04.565615v2-supplement-1.pdf]

bioRxiv preprint doi: https://doi.org/10.1101/2023.11.04.565615; this version posted February 15, 2024. The copyright holder for this preprint (which was not certified by peer review) is the author/funder, who has granted bioRxiv a license to display the preprint in perpetuity. It is made available under aCC-BY 4.0 International license.

Supplementary Materials

```
Data: Move table (M), current run index (i), current BWT offset (j)
Result: Index of run containing LF[j]
j ← M[i].π + (j − M[i].p)
i′ ← M[i].ξ
while M[i′].p + M[i′].n ≤ j′ do
|   i′ ← i′ + 1;
end
return i′
```

Algorithm S1: The fast forward algorithm, “ff”

```
Data: Move table (M), and pattern (p), number of runs (r), length of text (n)
Result: pml: pseudo matching lengths for the pattern P
ℓ ← 0, i ← r
j ← n
for k ← p.len to 1 do
|   c ← p[k]
|   if c ≠ M[i].c then
|   |   if (j − M[i].p) < M.Thresh[c] then
|   |   |   i ← RepositionUp(c, i)
|   |   else
|   |   |   i ← RepositionDown(c, i)
|   |   end
|   |   ℓ ← 0
|   end
|   pml[i] ← ℓ
|   i′ ← ff(M, i, j)
|   j′ ← M[i].π + (j − M[i].p)
|   i ← i′, j ← j′
|   ℓ ← ℓ + 1
end
return pml
```

Algorithm S2: PML computation using move structure. RepositionUp and RepositionDown are performed using scanning in the default mode, or the explicit pointers in the constant mode.

bioRxiv preprint doi: https://doi.org/10.1101/2023.11.04.565615; this version posted February 15, 2024. The copyright holder for this preprint (which was not certified by peer review) is the author/funder, who has granted bioRxiv a license to display the preprint in perpetuity. It is made available under aCC-BY 4.0 International license.

```
Data: Move table (M), and patterns (R), number of simultaneous reads to
      process(S)
Result: Generates all the PMLs for the patterns in R
Strands ← {}
for i ∈ S do
    s ← NextRead(R)
    Strands.add(s)
end
while hasReads(R) do
    for s ∈ Strands do
        NextPML(M, s)
        s.readPos ← s.readPos − 1
        if p.readPos < 0 then
            WritePMLs(s)
            s ← NextRead(R)
        else
            Prefetch(s.NextID)
        end
    end
end
```

Algorithm S3: The prefetching algorithm in move structure for computing PMLs. Each read is assigned to a class called "Strand" to be processed. After all the PMLs for a read in one Strand is computed, the Strand is updated to process the next read, "NextRead" retrieves the next read from the input file and assigns it to a strand. "NextPML" generates the pseudo matching length for the next base in the read assigned to strand s. "Prefetch" triggers an asynchronous retrieaval of the memory containing the destination row of the `LF`-mapping, which will be used later.

bioRxiv preprint doi: https://doi.org/10.1101/2023.11.04.565615; this version posted February 15, 2024. The copyright holder for this preprint (which was not certified by peer review) is the author/funder, who has granted bioRxiv a license to display the preprint in perpetuity. It is made available under aCC-BY 4.0 International license.

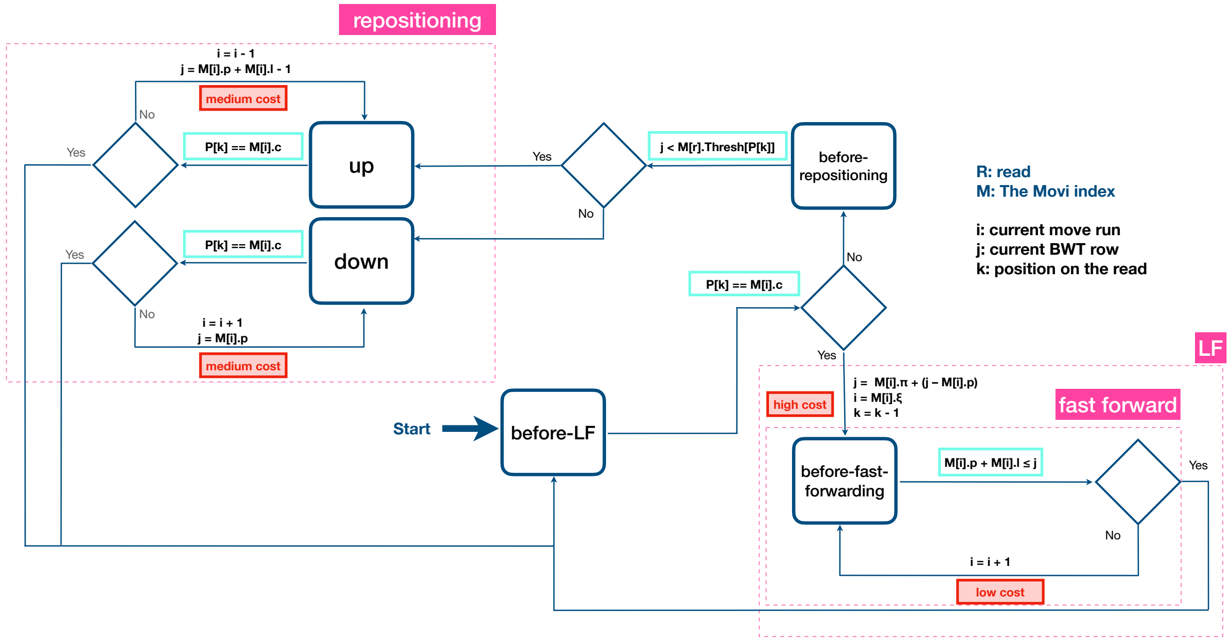

Figure S1: Full schematic of the pseudo matching length (PML) computation with Movi-default. For Movi-constant pointers and direct-address lookups are used instead of the linear-scan loops shown in the figure.

Mode	prefetch	size (GB)	query (hh:mm:ss)
default	no	8.5	00:45:22
constant	no	14	00:57:28
default	yes	8.5	**00:18:40**
constant	yes	14	00:24:01

Table S1: Comparison of different Movi modes. "Constant" refers to the mode that uses both (a) Nishimoto and Tabei's splitting procedure for ensuring a constant number of fast forwards and (b) constant-time direct-address lookups in the repositioning procedure. "Default" mode does not use splitting and uses linear scans in the repositioning procedure. "Prefetch" refers to whether Movi is using its latency-hiding approach. When "prefetch" is "yes," Movi is processing 16 reads concurrently and using prefetch instructions to hide cache-miss latency.

bioRxiv preprint doi: https://doi.org/10.1101/2023.11.04.565615; this version posted February 15, 2024. The copyright holder for this preprint (which was not certified by peer review) is the author/funder, who has granted bioRxiv a license to display the preprint in perpetuity. It is made available under aCC-BY 4.0 International license.

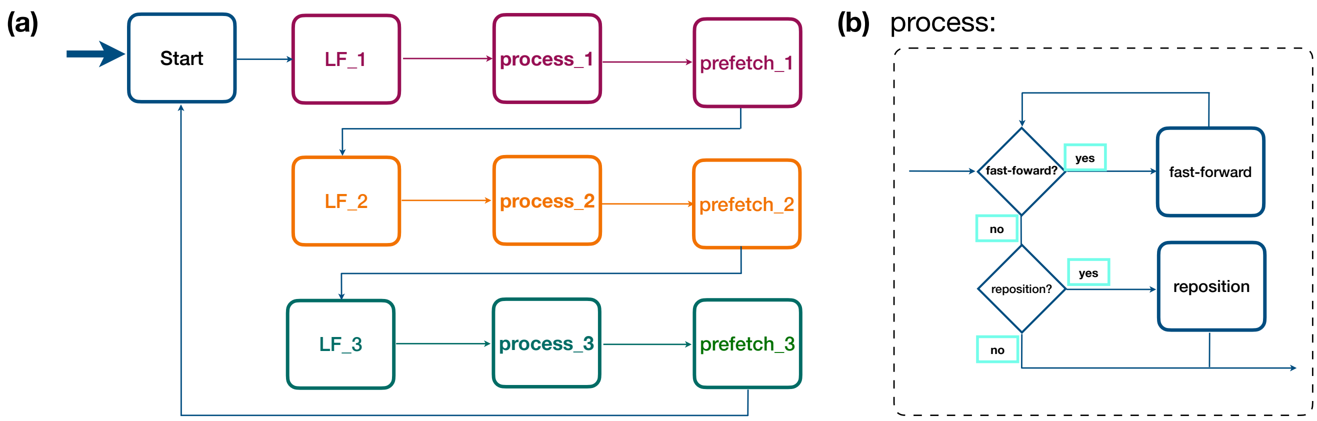

Figure S2: Computing PMLs with prefetching in Movi. (a) Shows how each read is processed until the LF step which is the highest cost. Then a prefetching step is triggered to fetch the memory required to do the LF. While the memory is being prefetched, the processor moves to processing another read for which the memory required is already prefetched, (b) The process which is performed after the LF for each read, note that all the steps in this processa are low cost or medium cost, therefore, these are much faster compared to the LF step for which the memory is prefetched.

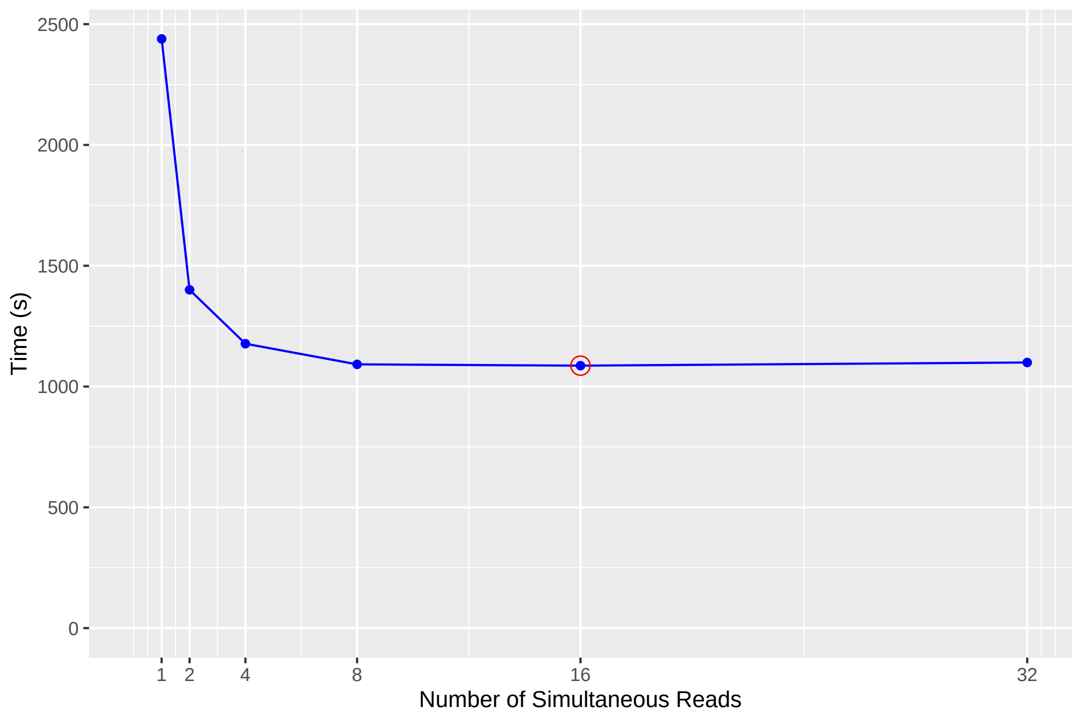

Figure S3: Time required to process the reads from the Zymo community using the latency-hiding strategy as a function of the number of reads being processed concurrently. The latency-hiding benefit accrues rapidly up to 8 threads, then plateaus. Movi processes 16 concurrent reads by default (red circle).

bioRxiv preprint doi: https://doi.org/10.1101/2023.11.04.565615; this version posted February 15, 2024. The copyright holder for this preprint (which was not certified by peer review) is the author/funder, who has granted bioRxiv a license to display the preprint in perpetuity. It is made available under aCC-BY 4.0 International license.

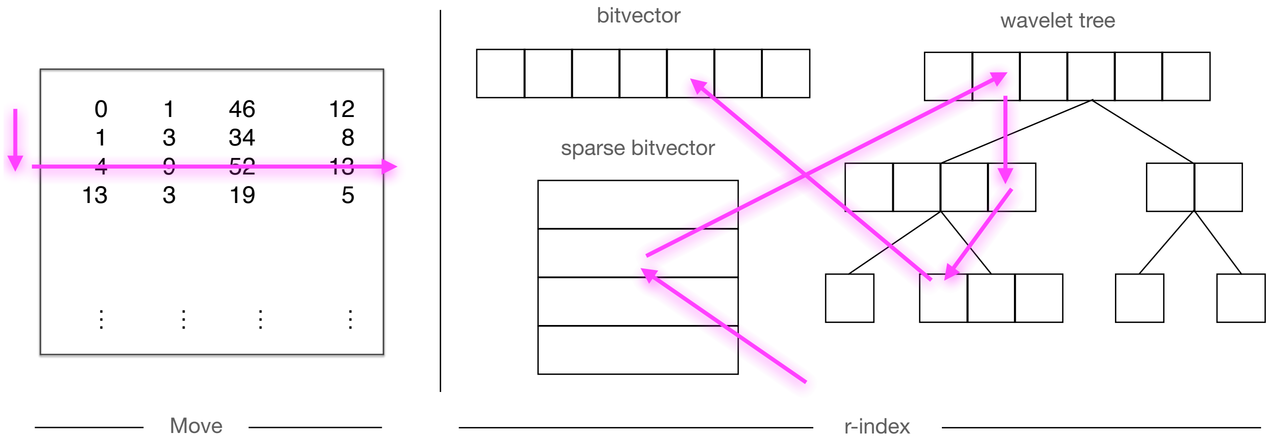

Figure S4: Approximate schematic for illustrating how memory accesses are induced for LF-mapping in r-index and move structure. This is not an exact representation of how queries work in these indexes and is just intended to roughly show the memory access patterns.

table for comparing the cache misses between movi and spumoni similar statistics are shown in sub-fig:cache

Tool	index (GB)		speed (10^6 base per second)		cache miss per base	
Movi	8.5	x4.7	11.29	x 30	1.61	-
SPUMONI	1.8	-	0.38	-	23.30	x 14.47

Table S2: cache miss comparison

operation	mode	mean	sd	max
fast-forward	Movi-default	0.4189303	3.682711	7694
	Movi-constant	0.3395261	0.913855	9
repositioning	Movi-default	1.039042	2.416862	4345
	Movi-constant	0.4068258	0.4912419	1

Table S3: Fast-forward and repositioning statistics for the Zymo sample.

mode	mean	sd	max	min
Movi-default	1.039042	2.416862	4345	0
Movi-constant	0.4068258	0.4912419	1	0
Movi-onebit	0.2719614	0.4724491	955	0
Movi-onebit-bv	0.2621271	0.4472	9	0

Table S4: Scans statistics.

bioRxiv preprint doi: https://doi.org/10.1101/2023.11.04.565615; this version posted February 15, 2024. The copyright holder for this preprint (which was not certified by peer review) is the author/funder, who has granted bioRxiv a license to display the preprint in perpetuity. It is made available under aCC-BY 4.0 International license.

sample	reference	Fulgor (hh:mm:ss)	SPUMONI (hh:mm:ss)	Movi (hh:mm:ss)
simulated (350K)	hprc 1	00:19:30	02:28:13	00:09:58
	hprc 94	00:22:39	02:38:12	00:17:19
combined (11M)	hprc 1	01:50:19	26:51:22	01:35:37
	hprc 94	02:04:21	28:31:58	01:44:17

Table S5: Query speed for the hprc dataset. The simulated sample consists of long reads simulated by PBSIM2[21] from a human genome. The combined sample consist of both the simulated reads and a human gut metagenomic sample (`SRR9847854`). The results for Movi in this table are evaluated without the latency hiding strategy.

Commands used for running the tools:

```
Building The indexes:
  $ spumoni build -i <reference_files_list> -o <index_prefix> -P -n
  $ fulgor build -l <reference_files_list> -o <output_prefix> -k 31 -m 19 -t 16
  $ minimap2 -x map-ont -d <index_file> <reference_file>
  $ bowtie2 --large-index <reference_file> <index_prefix>
Running the queries:
  $ spumoni run -r <index_prefix> -p <reads_file> -P -n
  $ fulgor -i <index_file> -q <reads_file> -o <output_file> -t 1
  $ minimap2 --secondary=no -t 16 -x map-ont <index_file> <reads_file> -o <output_file>
```
