# Supplementary material for: Movi: a fast and cache-efficient full-text pangenome index
Source: bioRxiv. 2023 Nov 5:2023.11.04.565615. Preprint. [Version 1] doi: 10.1101/2023.11.04.565615 (PMC10635132; doi:10.1101/2023.11.04.565615)
Supplement: Supplementary file 1 [file NIHPP2023.11.04.565615v1-supplement-1.pdf]

bioRxiv preprint doi: https://doi.org/10.1101/2023.11.04.565615; this version posted November 5, 2023. The copyright holder for this preprint (which was not certified by peer review) is the author/funder, who has granted bioRxiv a license to display the preprint in perpetuity. It is made available under aCC-BY 4.0 International license.

Supplementary Materials

Data: Move table (M), current run index (i), current BWT offset (j)
Result: Index of run containing LF[j]

```
j ← M[i].π + (j − M[i].p)
i′ ← M[i].ξ
while M[i′].p + M[i′].n ≤ j′ do
    i′ ← i′ + 1;
end
return i′
```

Algorithm S1: The fast forward algorithm, "ff"

Data: Move table (M), and pattern (p), number of runs (r), length of text (n)
Result: pml: pseudo matching lengths for the pattern P

```
ℓ ← 0, i ← r
j ← n
for k ← p.len to 1 do
    c ← p[k]
    if c ≠ M[i].c then
        if (j − M[i].p) < M.Thresh[c] then
            i ← RepositionUp(c, i)
        else
            i ← RepositionDown(c, i)
        end
        ℓ ← 0
    end
    pml[i] ← ℓ
    i′ ← ff(M, i, j)
    j′ ← M[i].π + (j − M[i].p)
    i ← i′, j ← j′
    ℓ ← ℓ + 1
end
return pml
```

Algorithm S2: PML computation using move structure. RepositionUp and RepositionDown are performed using scanning in the default mode, or the explicit pointers in the constant mode.

bioRxiv preprint doi: https://doi.org/10.1101/2023.11.04.565615; this version posted November 5, 2023. The copyright holder for this preprint (which was not certified by peer review) is the author/funder, who has granted bioRxiv a license to display the preprint in perpetuity. It is made available under aCC-BY 4.0 International license.

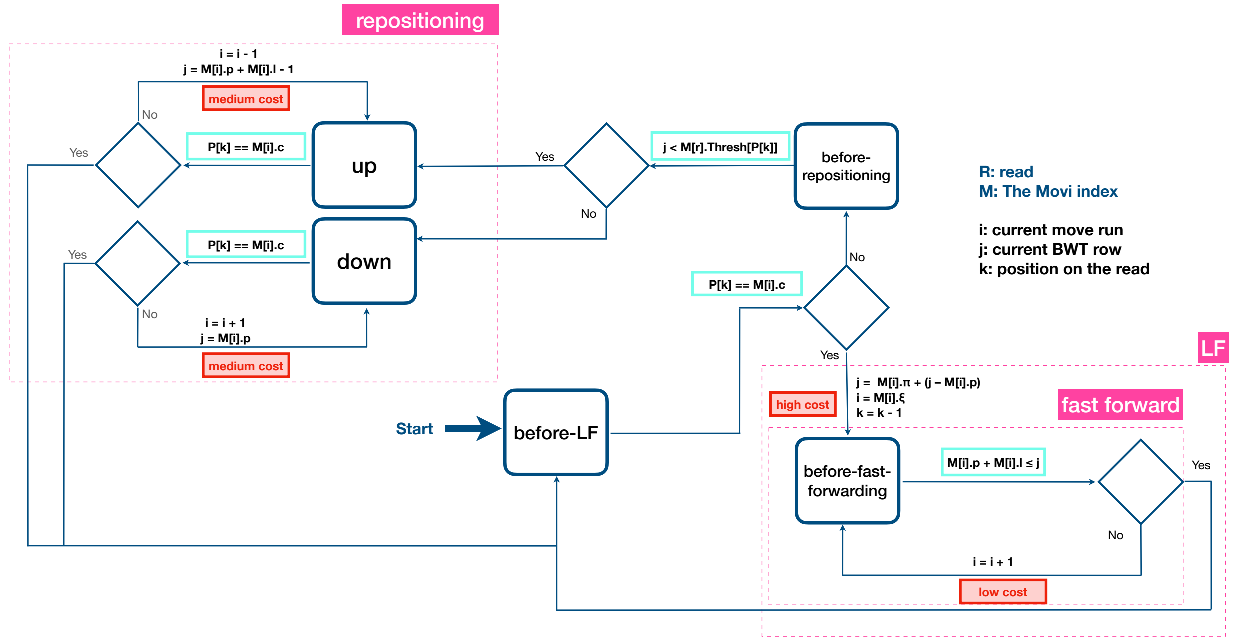

Figure S1: pseudo length computation with Movi-default. For Movi-constant pointers are used instead of the loops shown in the figure.

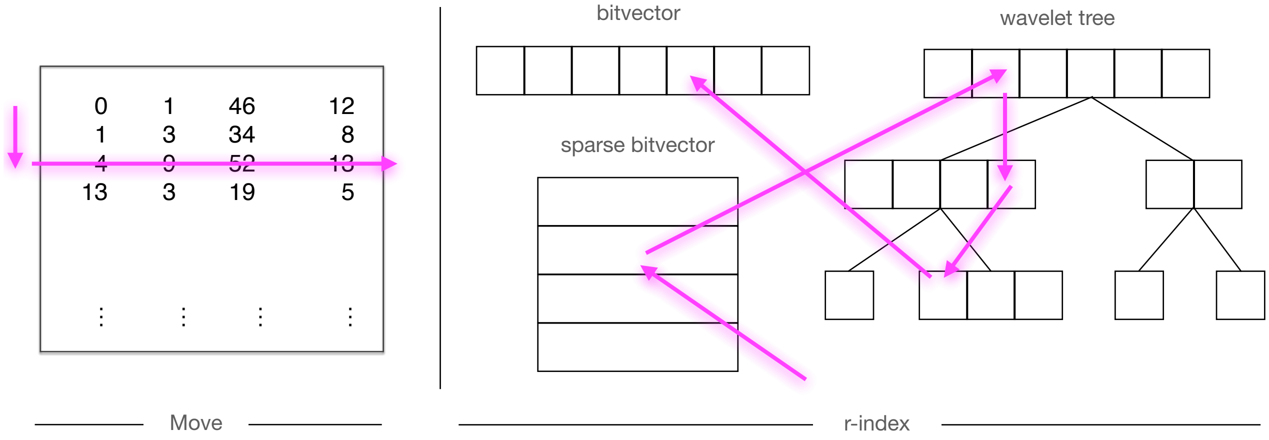

Figure S2: Approximate schematic for illustrating how memory accesses are induced for LF-mapping in r-index and move structure. This is not an exact representation of how queries work in these indexes and is just intended to roughly show the memory access patterns.

bioRxiv preprint doi: https://doi.org/10.1101/2023.11.04.565615; this version posted November 5, 2023. The copyright holder for this preprint (which was not certified by peer review) is the author/funder, who has granted bioRxiv a license to display the preprint in perpetuity. It is made available under aCC-BY 4.0 International license.

operation	mode	mean	sd	max
fast-forward	Movi-default	0.4189303	3.682711	7694
	Movi-constant	0.3395261	0.913855	9
repositioning	Movi-default	1.039042	2.416862	4345
	Movi-constant	0.4068258	0.4912419	1

Table S1: Fast-forward and repositioning statistics for the Zymo sample.

sample	reference	Fulgor (hh:mm:ss)	SPUMONI (hh:mm:ss)	Movi (hh:mm:ss)
simulated (350K)	hprc 1	00:19:30	02:28:13	00:09:58
	hprc 94	00:22:39	02:38:12	00:17:19
combined (11M)	hprc 1	01:50:19	26:51:22	01:35:37
	hprc 94	02:04:21	28:31:58	01:44:17

Table S2: Query speed for the hprc dataset. The simulated sample consists of long reads simulated by PBSIM2[20] from a human genome. The combined sample consist of both the simulated reads and a human gut metagenomic sample (`SRR9847854`).
